# EUS–guided liver biopsy: A useful and cost-effective alternative for specific indications in the study of liver diseases

**DOI:** 10.1097/eus.0000000000000155

**Published:** 2025-12-15

**Authors:** Teresa Alvarez-Nava, Carolina Ibarrola, Felipe de la Morena, José Díaz-Tasende, Yolanda Rodríguez-Gil, Carlos de la Serna, Cristina Martin-Arriscado, Ana Martín, Inmaculada Fernández, Ángel Sánchez, Mercedes Pérez-Carreras

**Affiliations:** 1 Gastroenterology Department, Biliopancreatic, Endoscopy and Hepatology Units, 12 de Octubre University Hospital, Madrid, Spain; 2 Faculty of Medicine, Complutense University, Madrid, Spain; 3 Pathology Department, University Hospital 12 de Octubre, Madrid, Spain; 4 Gastroenterology Department, University Hospital de la Princesa, Madrid, Spain; 5 Gastroenterology Department, University Hospital Río Hortega, Valladolid, Spain; 6 Scientific Support Unit, Research Institute (imas12), University Hospital 12 de Octubre, Madrid, Spain; 7 Department of Vascular Radiology, University Hospital 12 de Octubre, Madrid, Spain.

**Keywords:** Liver biopsy, EUS, Endohepatology, EUS–guided liver biopsy, Histological diagnosis, Metabolic dysfunction–associated steatotic liver disease, Cost-efficacy

## Abstract

**Background and Objectives:**

Comparative studies have demonstrated that EUS–guided liver biopsy (EUS-LB) is a useful and safe technique. However, there is not sufficient evidence information on their cost differences and potential applications. We aimed to investigate whether EUS-LB is a cost-effective alternative to traditional LB methods (percutaneous [PC-LB], transjugular [TJ-LB]) and determine its indications in clinical practice.

**Methods:**

In this observational, prospective, and multicenter study, patients who underwent EUS-LB at different tertiary centers (*N* = 52) were compared with a similar number of PC-LB (*N* = 50) and TJ-LB (*N* = 37) collected retrospectively. Diagnostic yield (percentage of conclusive histological diagnosis), specimen quality, adverse events, and cost-effectiveness were analyzed.

**Results:**

EUS-LB had 87% of diagnostic yield and 4% of mild adverse events, similar to traditional techniques (*P* = 0.097 and *P* = 0.252, respectively). Despite higher tissue fragmentation and lower longest specimen length in EUS-LB, no differences were found in the number of complete portal tracts, tissue adequacy (EUS-LB, 19%; PC-LB, 30%; TJ-LB, 36%; *P* = 0.164) or pathologist satisfaction, allowing adequate fibrosis stage assessment, particularly in metabolic dysfunction–associated steatotic liver disease. EUS-LB was more cost-effective when both LB and EUS were indicated (saving: €112.20 × 15% additional histological diagnosis [AHD]); in patients with cholestasis unsuitable for magnetic resonance cholangiopancreatography (MRCP) prior to LB; contraindication for the PC-LB, including cases of uncooperative individuals (saving: €234.75 × 15% AHD).

**Conclusions:**

EUS-LB is a useful alternative to traditional methods and the most cost-effective option when both LB and EUS are indicated, in cases of cholestasis as an alternative to MRCP and when the PC route is contraindicated.

## INTRODUCTION

Liver biopsy (LB) is still considered the gold standard for diagnosing liver conditions and providing prognostic information regarding the degree of inflammation and the stage of fibrosis of the liver lesion.^[1–3]^

Until about a decade ago, the most common procedure for collecting liver tissue specimens was percutaneous LB (PC-LB), with transjugular LB (TJ-LB) being used only when PC-LB was contraindicated.^[4,5]^ However, the recent development of interventional EUS and its application to liver disease, a highly specialized field termed endohepatology,^[6–10]^ now allows specimen collection from both liver lobes, with a linear echoendoscope guiding the needle from the gastric and duodenal stations, in real time and without interference from other structures.^[11–13]^

Since Mathew^[14]^ reported the first EUS-guided LB (EUS-LB), several studies, systematic reviews, and meta-analyses have been published confirming its efficacy and safety comparing it with traditional LB methods. However, most of them are single-center retrospective studies, which are very heterogeneous given the lack of protocolization of the procedure.^[15–21]^ Moreover, the histopathological criteria for specimen adequacy are not standardized, and the pathologist’s ability to diagnose and stage liver disease is not considered.^[1,22–29]^

Information on the cost-effectiveness of this new LB technique is limited. Although EUS-LB requires two simultaneous procedures and deep sedation of the patient, EUS and LB can be performed in the same procedure when both techniques are indicated.^[10,13,22,30]^

Our aim was to determine whether EUS-LB is an effective, safe, and cost-effective alternative LB method in a multicenter prospective cohort study, comparing it with traditional methods. We also aim to familiarize clinicians and pathologists with this new LB technique, including a proposal for specific indications.

## METHODS

### Study design and patient selection

We conducted a multicenter prospective cohort study involving patients who underwent EUS-LB at several tertiary hospitals in our country (July 2017–February 2021), which was approved by the institutional review board (reference number 17/092) at the hospital coordinating the study (12 de Octubre University hospital, HU12O). Patients aged >18 years with an indication for LB and any of the following conditions were included: contraindication for PC-LB (severe obesity, massive ascites or liver focal lesions, lack of cooperation); cholestatic pattern of liver enzymes, performing EUS-LB once extrahepatic causes have been ruled out by simultaneous biliopancreatic EUS; and prescription of EUS for any indication. In these cases, both procedures were performed simultaneously. Pregnant patients and those with coagulopathy or anticoagulant and/or antiplatelet drugs not discontinued (according to current recommendations for endoscopic procedures)^[31]^ were excluded.

To compare the EUS-LB cases with those involving traditional LB methods, a similar number of PC-LBs and TJ-LBs performed at HU12O during the same study period were retrospectively collected.

### Description of the LB procedures

#### EUS-guided LB

**Figure 1:**
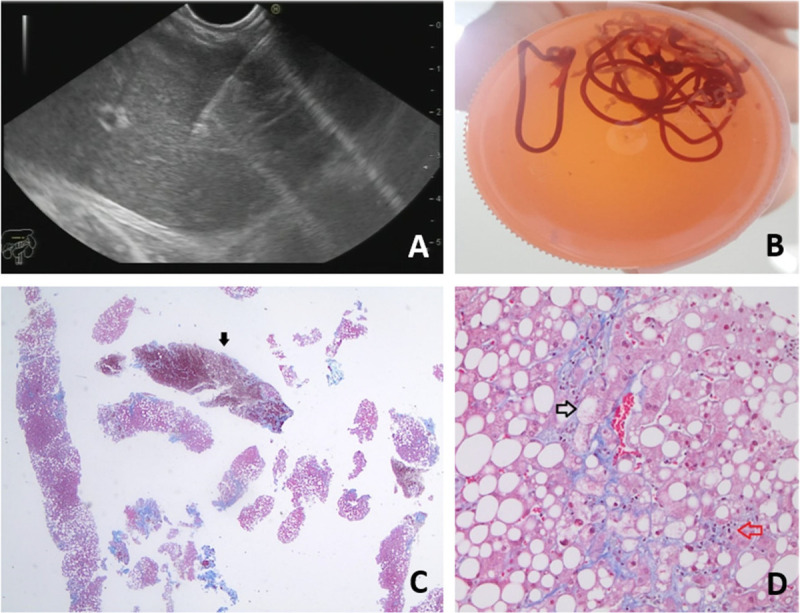
Core biopsy specimen obtained by EUS-LB (EUS–guided liver biopsy): (A) EUS-LB of the left hepatic lobe; (B) core biopsy specimen in formalin; (C) photomicrograph showing multiple fragments of liver tissue and blood clots (arrow); (D) diagnosis of steatohepatitis with stage 1 fibrosis (Masson trichromic stain): intense macrovacuolar steatosis and prominent hepatocyte ballooning (black arrow) with inflammatory lobular infiltrates (red arrow).

The procedure was standardized by following a protocol that established that one or two experienced endosonographers from each center would perform EUS and LB using a linear endoscope attached to an ultrasound console (Pentax EG-3870UTK, Germany; Olympus GF-UCT-180, Japan). A 19-gauge fine-needle aspiration (FNA-19G) cytology needle (Boston Scientific Expect™ Slimline, Cook Medical EchoTip® Ultra) was used. Two transgastric punctures were made on the left liver lobe (LLL), with the probe positioned immediately distal to the cardia and rotated counterclockwise to access the liver segments II and III [Figure 1A]. If the collected specimen was insufficient, at the endosonographer’s discretion, a third puncture was performed on the right liver lobe (RLL) from the duodenal station. In all cases, the needle penetrated 2–6 cm into the liver parenchyma, with 7–10 to-and-fro movements per puncture. Dry or wet suction with saline solution and fanning needle movements were used at the endosonographer’s decision. Thanks to real-time visualization, vascular structures (color Doppler ultrasound) and intrahepatic bile ducts were avoided in the path of the needle. The collected liver tissue was immediately embedded in 10% formalin by slowly and carefully pushing it with the stylet to prevent fragmentation of the specimen [Figure 1B]. LLL and RLL specimens were placed in different containers, labeled, and sent to the dedicated liver pathologists at the Department of Anatomical Pathology at each participating center. The EUS-LB procedure and the type of collected specimen did not allow the pathologist to be blinded to the LB method. In the anatomical pathology laboratory, the biopsy tissue was carefully separated from the clots for subsequent independent processing of tissue cylinders >5 mm and the rest of the material pooled together. Standard staining procedures were performed [Figures 1C, D].

#### PC-guided LB

With the patient in left lateral decubitus position and under local anesthesia, a small cut was made in the skin in an area affording the best access to the RLB, previously located and marked by conventional ultrasound (Toshiba Aplio XG iStyle Ultrasound). LB was performed by a hepatologist using a 16G needle according to the standard technique.

#### TJ-guided LB

The specimens were collected by an interventional radiologist who accessed the right hepatic vein from the internal jugular vein under fluoroscopic guidance, performing the LB on the RLL using a 18G Tru-Cut biopsy needle.

PC-LB and TJ-LB specimens were embedded in 10% formalin and sent for histological analysis.

### Variables evaluated

The following variables were evaluated: demographics (sex and age); related to the LB procedure: indication, type of suction, unilobar or bilobar biopsy, adverse events (AE), resolution, and recovery time; histological: number of complete portal tracts (CPT, defined as either one in which the complete circumference of the portal tract was visible within the tissue fragment and which contained at least two of three portal structures, namely, the bile duct, portal vein, or hepatic artery, or one in which at least 80% of the circumference was visible within the tissue fragment and which contained at least two of three portal structures),^[27]^ longest specimen length (LSL), number of tissue fragments, histological diagnosis, specimen adequacy (according to the criteria of the European Association for the Study of the Liver-EASL: LSL >15 mm, >6 CPT)^[3,5]^; satisfaction of the pathologist; and clinical impact of the histological diagnosis (understood as repercussions on medical decisions).

For EUS-LB, intraoperative complications were assessed immediately, and subsequent complications were evaluated during an observation period of 1–3 hours at the day hospital of the Endoscopy Unit. Late AEs were collected via phone calls 24–72 hours and 30 days after the procedure.

Intraoperative and postoperative complications, as well as AE related to PC-LB and TJ-LB, were collected from data stored in the electronic medical records. All specimens collected by PC-LB and TJ-LB were retrieved from the histological specimen repository of the Department of Anatomical Pathology and reviewed by HU12O pathologists prior to inclusion.

### Cost effectiveness analysis

The time frame considered for the cost-effectiveness analysis was 2017–2024. A health care and hospital perspective is assumed as a centerpiece in the realization of the study.

**Figure 2:**
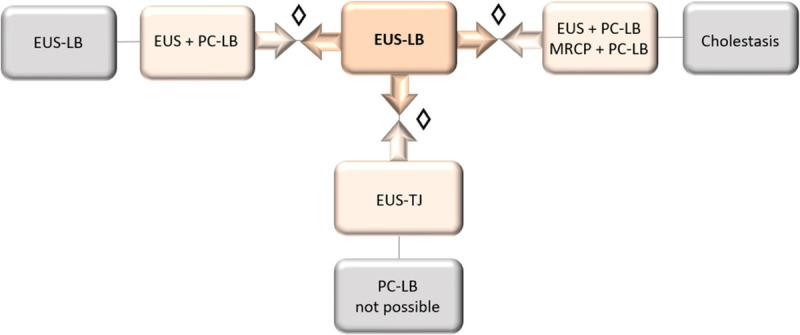
Flowchart of the comparative cost-effectiveness analysis of EUS–guided liver biopsy (EUS-LB) *versus* traditional methods: PC (percutaneous)-LB and TJ (transjugular)-LB. ◊ Comparison of methods according to indication for EUS-LB (gray box). Magnetic resonance cholangiopancreatography (MRCP).

Efficacy was measured using the percentage of histological diagnoses (diagnostic yield) obtained by each of the three techniques that were compared in our study. The direct costs were calculated using data extracted from official documents of the public health system of Autonomous Region of Madrid and the catalogue of the Spanish Society of Medical Radiology [Table 1]. The indirect costs, such as anesthesia, recovery period, and fees of nurses and physicians involved in each procedure, were obtained from the database of year-end closing process for analytical accounting of the HU12O [Table 1]. Based on this, we performed a comparative analysis of overall cost-effectiveness and a subanalysis according to the indication for which EUS-LB was requested. For cholestasis as an indication, the costs included the alternative EUS *versus* MRCP to rule out biliary obstruction before performing LB [Figure 2].

**Table 1:** Cost calculation of each procedure using data extracted from official documents of the public health system of Madrid’s region, the catalogue of the Spanish Society of Medical Radiology, and the year-end closing process for analytical accounting of the 12 de Octubre University Hospital.

PRICE (euros)	EUS-LB	PC-LB	PC-LB + MRCP	PC-LB + EUS	TJ-LB
Stance in day hospital*	10.60	85.00	85.00	95.60	
Analysis of the sample in anatomic pathology				
Madrid 2017*	104.00	104.00	104.00	104.00	104.00
Madrid 2024*	104.00	104.00	104.00	104.00	104.00
Andalucía^†^	62.45	62.45	62.45	62.45	62.45
Hepatic puncture					
Madrid 2017^‡.§^	145.00	145.00	145.00	145.00	744.32
Madrid 2024^§,∥^	145.00	145.00	145.00	145.00	744.32
Andalucía^†^	310.87	282.64	282.64	282.64	2191.84
EUS					
Madrid 2017^‡^	277.00			277.00	
Madrid 2024^∥^	527.00			527.00	527.00
Andalucía^†^	631.00			631.00	
Inpatient bed*					212.35
Specialist physician*	68.92	34.46	34.46	103.38	34.46
Nurse*	39.98	19.99	19.99	59.97	19.99
Nursing assistant*	14.01	14.01	14.01	28.02	14.01
MRCP					
Madrid 2017^‡^			180.00		
Madrid 2024^∥^			199.00		
Andalucía^†^			175.35		
Total price (euros)					
Madrid 2017	659.51	402.46	582.46	812.97	1129.13
Madrid 2024	909.51	402.46	601.46	1062.97	1129.13
Andalucía	1137.83	498.55	673.9.	1263.06	2535.10

Comparison with other Spanish public health regions.

EUS-LB: EUS–guided liver biopsy; PC-LB: Percutaneous liver biopsy; MRCP: Magnetic resonance cholangiopancreatography; TJ-LB: Transjugular liver biopsy.

Data extracted from *2020 accounting closure of the 12 de Octubre University Hospital, from the ^†^BOCM (Official Gazette of Madrid’s region) in force during the time period of our study (BOCM issue 198, pages 7–39, Monday, August 21, 2017), from the ^‡^2016 SERAM (Spanish Society of Medical Radiology) catalogue (Coste URV*—*Relative Value Units or relative resource utilization weight of each scan), from the ^§^most recent BOCM (order 1975, pages 1–123. December 29, 2023), and from the ^∥^BOJA (Official Gazette of the Andalusian Regional Government, number 108, pages 45904/1-163, June 5, 2024).

To evaluate the robustness of our cost-effectiveness analysis across regional and temporal cost variations, we performed a sensitivity analysis using prices from another national region (Public Health System of Andalucía) and historical costs from our own region [Table 1].

### Statistical analysis and sample size calculation

Based on Rothman and Greenland,^[32]^ assuming a diagnostic efficacy of 90% for PC-LB (the gold standard) and 85% for EUS-LB, with a type I error (*α*) of 0.1 and a 90% confidence interval, a sample size of 47 participants was determined. To account for potential dropouts, this number was rounded to 50. For the comparative analysis, an equal number of patients who underwent traditional procedures at HU12O during the study period were randomly and retrospectively selected.

The different variables were compiled into a single database at the coordinating hospital of the study (HU12O). SPSS (version 25) program was used for statistical analyses. Variables were described using mean with standard deviation, range, or percentage. Pearson chi-square was used to compare the procedural characteristics in the EUS-LB, PC-LB, and TJ-LB cohorts, applying the Bonferroni method for comparisons of two techniques. *P* < 0.05 was considered statistically significant. A multilevel logistic regression analysis of the demographic characteristics and histological diagnosis was performed. For the cost-effectiveness analysis, a deterministic model was developed using a decision tree according to the indication for the LB technique [Figure 2]. The results are expressed as the incremental cost-effectiveness ratio (ICER) in euros for an additional histological diagnosis (AHD) of 1% and 15% between the two procedures.

## RESULTS

Our study included 52 patients who underwent EUS-LB between July 2017 and February 2021. For comparison, data were retrospectively collected from the electronic medical records at HU12O, including 50 randomly selected PC-LB patients and 37 consecutively included TJ-LB patients, representing the full cohort of TJ-LBs performed during the study period.

Table 2 summarizes the indications for EUS-LB, variables related to the procedure, and histological diagnoses. Comparative analysis of EUS-LB with retrospective cohorts of traditional methods found no differences in demographic characteristics, diagnostic yield, histological diagnoses, or AE [Table 3]. The multilevel logistic regression showed no differences in the relative risk ratio between the demographic characteristics and histological diagnosis of the three cohorts [Table 4].

**Table 2:** Indications, procedure-related variables, and histological diagnoses in 52 patients using EUS–guided liver biopsy.

Indication for EUS-LB			
1. Cholestasis without obstruction of the biliary tract: 59% (31/52)		
2. EUS + LB: 33% (17/52)		
3. PC-LB not recommended: contraindicated (ascites, FLL) 4%(2/52); lack of cooperation 4% (2/52)			
Biopsied liver lobe	Both lobes: 71% (37/52)	LLL: 71% (37/52)	*P*-value
Diagnostic yield	95% (35/37)	67% (10/15)	**0.008**
>6 CPT and LSL >15 mm	24% (9/37)	7% (1/15)	0.143
>6 CPT	78% (29/37)	33% (5/15)	**0.002**
Type of suction	Wet: 77% (40/52)	Dry: 23% (12/52)	*P*-value
Diagnostic yield	93% (37/40)	67% (8/12)	***0.021***
>6 CPT and LSL >15 mm	23% (9/40)	1% (1/12)	*0.275*
>6 CPT	73% (29/40)	42% (5/12)	*0.049*
Histological diagnoses			
1. MASLD: 34% (15/45) 2. Minimal changes: 20% (9/45) 3. DILI: 14% (6/45) 4. Alcoholic steatohepatitis: 9% (4/45) 5. Autoimmune hepatitis: 9% (4/45) 6. Primary biliary cholangitis: 4% (2/45) 7. Primary sclerosing cholangitis: 2% (1/45) 8. Multiple biliary hamartomas: 2% (1/45) 9. Hemochromatosis: 2% (1/45) 10. Granulomatous hepatitis: 2% (1/45) 11. Metabolic disorders: 2% (1/45)		

Significant *P*-value <0.05 in bold font.

LB: Liver biopsy; FLL: Focal liver lesion; LLL: Left liver lobe; CPT: Complete portal tracts; LSL: Longest specimen length; MASLD: Metabolic-associated steatotic liver disease; DILI: Drug-induced liver injury.

**Table 3:** Comparison of demographics, diagnostic yield, clinical impact, and complications of EUS–guided liver biopsy *vs*. traditional methods.

Technique	EUS-LB (*N* = 52)	PC-LB (*N* = 50)	TJ-LB (*N* = 37)
Age (yr) (*P* = 0.087)	54 ± 13	49 ± 16	55 ± 18
Women (*P* = 0.286)	63% (33/52)	48 (24/50)	54% (20/37)
Diagnostic yield (*P* = 0.097)	87% (45/52)	92% (46/50)	76% (28/37)
Most frequent histological diagnoses (*P* = 0.001)	1. MASLD: 34% (15/45) 2. Minimal changes: 20% (9/45) 3. DILI: 14% (6/45)	1. Minimal changes: 24% (11/46) 2. Liver graft rejection: 15% (7/46) 3. Autoimmune hepatitis: 13% (6/46)	1. Autoimmune hepatitis: 35% (10/28) 2. DILI: 21% (6/28) 3. Liver graft rejection: 14% (4/28)
Clinical impact (*P* = 0.016)	88% (46/52)	94% (47/50)	73% (27/37)
Complications (*P* = 0.252)			
Proportion	4% (2/52)	10% (5/50)	15% (3/37)
Type	Abdominal pain: 1/2 (2%) Asymptomatic pneumobilia: 1/2 (2%)	Bleeding: 2/5 (4%) Abdominal pain: 2/5 (4%) Fever: 1/5 (2%)	Abdominal pain: 1/3 (5%) Fever: 1/3 (5%) Bleeding: 1/3 (5%)
Chronology	Late (≥24 h): 1/2 (2%) Intraoperative: 1/2 (2%)	Early (<24 h): 5/5 (10%)	Late (≥24 h): 2/3 (10%) Early (<24 h): 1/3 (5%)
Treatment	Conservative management: 2/2 (4%)	Urgent radiological intervention: 2/5 (4%) Conservative management: 3/5 (6%)	Conservative management: 2/3 (10%) Urgent radiological intervention: 1/3 (5%)

EUS-LB: EUS–guided liver biopsy; PC-LB: Percutaneous liver biopsy; TJ-LB: Transjugular liver biopsy; MASLD: Metabolic dysfunction–associated steatotic liver disease; DILI: Drug-induced liver injury.

**Table 4:** Univariate multilevel logistic regression analysis of age, sex, and histologic diagnoses of the prospective cohorts of EUS–guided liver biopsy (whose values are taken as reference for the analysis) *vs*. the traditional technique groups.

	PC-LB	TJ-LB
	RRR (CI 95%)	
Age	0.98 (0.95–1.00)	1.00 (0.98–1.03)
Male sex	1.88 (0.85–4.15)	1.48 (0.63–3.48)
Histological diagnosis		
PBC	1.64 (0.24–11.08)	
PSC	1.64 (0.13–21.11)	
AH	1.23 (0.26–5.73)	11.25 (1.65–76.85)*
DILI	0.14 (0.01–1.35)	4.05 (0.67–30.23)
MASLD	0.27 (0.07–1.04)	0.29 (0.02–3.79)
GH	0.82 (0.04–14.99)	1.12 (0.08–16.30)

For sex, the values of the females sex were taken as reference; for histological diagnoses, the values of the minimal diagnostic changes were taken as reference.

RRR: Relative risk ratio; CI: Confidence interval; PC-LB: Percutaneous liver biopsy; TJ-LB: Transjugular liver biopsy; PBC: Primary biliary cholangitis; PSC: Primary sclerosing cholangitis; AH: Autoimmune hepatitis; DILI: Drug-induced liver injury; MASLD: Metabolic dysfunction–associated steatotic liver disease; GH: Granulomatous hepatitis.

*Very wide range, result not interpretable.

As shown in Table 3, only 2/52 patients (4%) experienced AE related to EUS-LB (mild abdominal pain after 24 hours and asymptomatic intraoperative pneumobilia), which resolved with conservative management. For traditional methods, 10% (5/50) and 8% (3/37) of patients experienced AE after PC-LB and TJ-LB, respectively, with pain, fever, and immediate bleeding occurring most frequently (2/5 patients in the PC-LB group and 1/3 in the TJ-LB group required urgent radiological intervention). The recovery time was 1 hour in EUS-LB, 6–8 hours in PC-LB, and 24 hours in TJ-LB.

The clinical impact of diagnoses obtained with EUS-LB was similar to that of PC-LB and higher than that of TJ-LB [Table 3].

**Figure 3:**
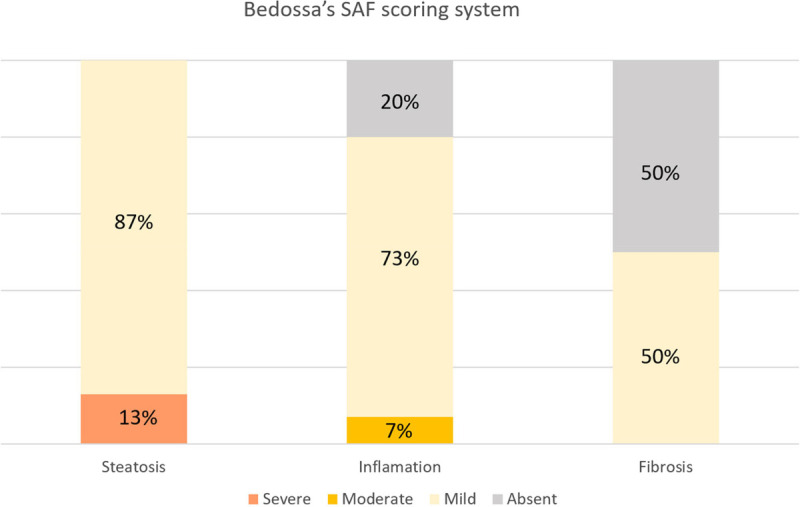
Characteristics of the 15 patients with MASLD diagnosed by EUS-LB. MASLD: Metabolic dysfunction–associated steatotic liver disease; EUS-LB: EUS–guided liver biopsy; SAF: Steatosis-Activity-Fibrosis.^[33]^

Metabolic dysfunction–associated steatotic liver disease (MASLD) was the most frequent diagnosis in the EUS-LB group. EUS-LB allowed not only a histopathological diagnosis but also the description of liver lesions, including fibrosis, using Bedossa’s Steatosis-Activity-Fibrosis (SAF) scoring system [Figure 3].^[33]^

The histological findings and the grade of satisfaction of the pathologist for the three LB techniques are shown in Table 5. Specimens collected by EUS-LB were more fragmented and therefore had lower LSL, although the number of CPT and the percentage of adequate samples were similar in the three techniques.

**Table 5:** Comparison of histological variables according to EASL criteria^[3]^ and the degree of satisfaction of the pathologist for the three liver biopsy techniques.

	EUS-LB (*n* = 52)	PC-LB (*n* = 50)	TJ-LB (*n* = 37)
Number of fragments of the specimen (*P* < 0.001)	9 ± 2	2 ± 1	4 ± 3
LSL (mm) (*P* < 0.001)			
Mean	10 ± 8	15 ± 7	14 ± 8
Range	1–50	4–30	5–45
Number of CPTs (*P* = 0.571)			
Mean	9 ± 8	7 ± 3	8 ± 5
Range	0–34	1–20	0–21
Adequate specimen			
>6 CPT (*P* = 0.985)	34/52 (67%)	24/37 (65%)	33/50 (66%)
>6 CPT and LSL >15 mm (*P* = 0.164)	10/52 (19%)	11/37 (30%)	18/50 (36%)
Pathologist satisfaction (*P* = 0.117)			
Good	58% (30/52)	82% (41/50)	70% (26/37)
Acceptable	25% (13/52)	12% (6/50)	16% (6/37)
Poor	17% (9/52)	6% (3/50)	14% (5/37)

EUS-LB: EUS–guided liver biopsy; PC-LB: Percutaneous liver biopsy; TJ-LB: Transjugular liver biopsy; LSL: Longest specimen length; CPT: Complete portal tracts.

**Figure 4:**
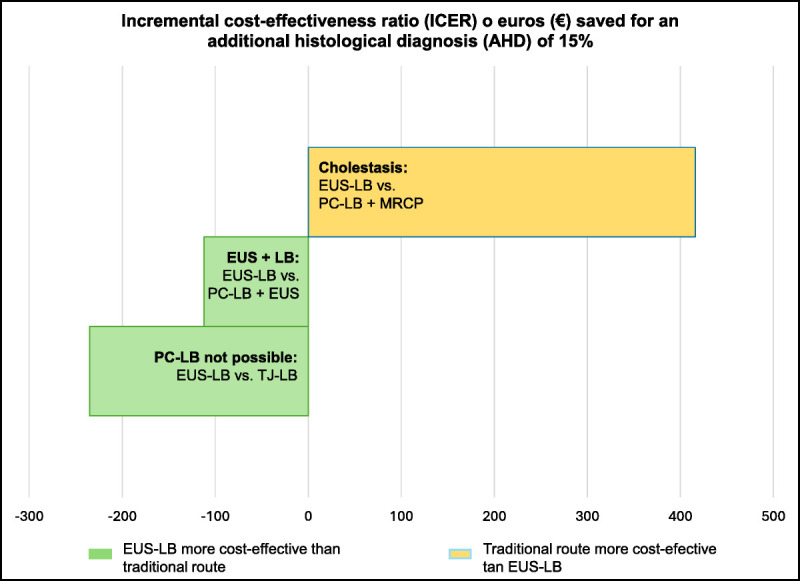
Cost-effectiveness analysis according to the indications for EUS–guided liver biopsy. The graphic shows an ICER increase or euros (€) saved per 15% of AHD obtained with EUS-LB when compared with traditional methods. ICER: Incremental cost-effectiveness ratio; AHD: additional histological diagnosis; PC-LB: Percutaneous liver biopsy; MRCP: Magnetic resonance cholangiopancreatography; LB: Liver biopsy; EUS-LB: EUS–guided liver biopsy; TJ-LB: Transjugular liver biopsy.

Table 6 and Figure 4 show the overall cost-effectiveness analysis of EUS-LB compared with the two traditional methods and according to each of the three indications for EUS-LB included in our study. PC-LB was the most cost-effective method when the indication was only LB (€501.30 saved with PC-LB *vs*. EUS-LB for each 15% AHD) and EUS-LB when the patient required EUS in addition to LB (€112.20 saved for each 15% AHD). According to our data, in cases of cholestasis, MRCP and PC-LB are more cost-effective than EUS-LB with biliary tract exploration in the same procedure (€415.65 saved when compared to EUS-LB for each 15% AHD). In contrast, when PC-LB was contraindicated, EUS-LB was more cost-effective than TJ-LB (€234.75 saved compared to TJ-LB for each 15% AHD). The sensitivity analysis confirmed that the cost-effectiveness outcomes remained consistent despite variations in economic sources across different time periods and health regions, supporting the robustness of our findings.

**Table 6:** Overall cost-effectiveness study analysis according to indications for EUS-LB and sensitivity analysis to assess variability in costs between public health systems in different regions and economic changes over time.

	Overall analysis (EUS-LB *vs*. PC-LB *vs*. TJ-LB)				Analysis by indication	
					Cholestasis	EUS + LB	PC-LB not possible or contraindicated
		EUS-LB *vs*. PC-LB	PC-LB *vs*. TJ-LB	EUS-LB *vs*. TJ-LB	EUS-LB *vs*. PC-LB + MRCP	EUS-LB *vs*. EUS + PC-LB	EUS-LB *vs*. TJ-LB
Efficacy difference	7.69	19.74	12.05	2.78	20.51	30.00
Cost difference						
Madrid 2017	257.05	−726.67	−469.62	77.00	−153.46	−469.51
Madrid 2024	507.05	−726.67	−219.62	308.05	153.46	−219.62
Andalucía	639.28	−2036.55	−1397.27	589.16	125.23	−1272.04
ICER or € saved per each percentage of AHD					
Madrid 2017	33.42	−36.81	−38.97	27.71	−7.48	−15.65
Madrid 2024	65.94	−36.81	−18.23	110.81	−7.48	−7.32
Andalucía	83.13	−103.17	−115.96	211.93	−6.11	−42.40

EUS-LB: EUS–guided liver biopsy; PC-LB: Percutaneous liver biopsy; TJ-LB: Transjugular liver biopsy; MRCP: Magnetic resonance cholangiopancreatography; ICER: Incremental cost-effectiveness ratio; €: Euros; AHD: Additional histological diagnosis.

## DISCUSSION

Several studies have confirmed the efficacy and safety of EUS-LB.^[14,25,28]^ This new access route to the liver has additional advantages when compared with traditional LB methods, as it facilitates the guiding of the needle with real-time standard and Doppler ultrasound (thereby avoiding vessels and ducts). It facilitates collecting specimens from both liver lobes and performing multiple interventions in the parenchyma with a single puncture of the liver capsule,^[12,13]^ with the patient under sedation, resulting in a shorter recovery time and fewer AE.^[18,21]^ Despite all this, most hepatologists perceive EUS-LB as an endoscopic technique and trust the traditional LB methods more, mainly PC-LB, using TJ-LB when PC-LB is contraindicated.^[1,4,5]^ Pathologists also do not appear to feel comfortable analyzing the samples obtained by EUS-LB.^[27]^

Our study was carried out by a multidisciplinary group of investigators with the aim of providing practical insights into what EUS-LB can bring to the gastroenterology and hepatology community. To this end, our study also analyzed pathologist satisfaction, the efficacy of EUS-LB in some specific liver diseases such as MASLD, and its cost-effectiveness compared to traditional LB methods, in order to find the most useful scenarios for this new technique.

Unlike most studies where abnormal liver enzyme levels are the primary indication for EUS-LB,^[28]^ our study considered EUS-LB as an alternative to the traditional PC-LB from the beginning. Therefore, we included patients requiring imaging (such as MRCP or EUS) to rule out bile duct obstruction prior to liver biopsy; those with an indication for EUS—typically due to subepithelial or cystic pancreatic lesions—alongside the need for liver biopsy; and patients with contraindications to the percutaneous route. This approach was the one proposed in the first published series and seems more practical when analyzing the cost-utility of EUS-LB and its applicability in clinical practice.^[34]^

Overall, EUS-LB provides a diagnostic yield of 93%–94% and is associated with 2.3% of AE, with serious complications, such as hemorrhage and subcapsular hematoma after puncture, occurring very rarely.^[15,16]^ Our series of 52 EUS-LBs had a diagnostic yield of 87% and 4% AE (one patient with mild, self-limited abdominal discomfort, and another with asymptomatic intrahepatic pneumobilia). Although the percentage of diagnoses in our study is lower compared to other publications,^[17,18]^ it is important to consider it in the context of other variables, including clinical impact, specimen adequacy, and satisfaction of the pathologist with the specimen.

Very few studies have assessed the performance of EUS-LB in terms of clinical impact.^[35]^ In our series, 100% of the histological diagnoses obtained by EUS-LB were consistent with the clinical scenario and had an impact on subsequent patient management. The clinician did not consider repeating the LB using a different access route in any of the cases.

According to the protocol established with the pathologists in our study, most specimens had >10 fragments, and only 19% were adequate (67% if considering only the >6 CPT criteria). This is probably because, unlikely most authors,^[19,22,35]^ our study considered the length of the longest fragment and not the total specimen length, sum of all fragments, because it reflects better the preserved architecture.^[25,27]^ Besides, one must take into account that the reduction of the specimen’s length and diameter produces a significantly lower number of CPT and a higher number of incomplete portal tracts in a single core.^[25,26]^ Nevertheless, this may potentially be compensated by the greater number of tissue pieces and fragments obtained with the EUS-LB.^[27]^ Despite all this, pathologists rated their satisfaction with the specimen as good (58%) or acceptable (27%) for establishing the histological diagnosis and prognosis in most cases, considering both inflammation and fibrosis.

Evidence on the efficacy of EUS-LB for some specific indications is limited. This is of special importance in parenchymal liver diseases as prevalent as MASLD in which the histological lesion is mostly found in the centrilobular areas, possibly reducing artifacts due to fragmentation of the specimen.^[30,36,37]^ The most frequent histological diagnosis in our series of EUS-LB was MASLD (34%), with the stage of fibrosis, severity of steatosis, and degree of inflammation satisfactorily established in almost all cases [Table 3; Figure 3].

Although it was not an objective, some variables related to the EUS-LB procedure that can improve the diagnostic yield and quality of the specimen have been analyzed.^[38–41]^ In our study, the diagnostic yield and the quality of the specimen were much higher using wet suction and performing bilobar biopsy [Table 2].

Several studies comparing EUS-LB with PC-LB and TJ-LB have been conducted since 2015,^[40–42]^ although most are retrospective and included in systematic reviews and meta-analyses.^[15–20]^ Recent prospective randomized controlled studies comparing EUS-LB with PC-LB have been carried out.^[22,35,43]^

Consistent with the results reported by Pineda et al.,^[42]^ our multicenter prospective EUS-LB series was compared with a similar number of retrospective PC-LB and TJ-LB cases. Those authors found that specimens obtained by EUS-LB, especially when collected from both liver lobes, were adequate in 98% of cases and comparable to those of PC-LB or TJ-LB procedures. Nakanishi et al.^[27]^ focused their discussion of the results from 113 EUS-LB on the histological aspects of this type of specimen and compared them retrospectively with a similar number of PC-LB and TJ-LB cases. For these authors, the specimen was adequate when it allowed the diagnosis and staging of liver disease, whether it met the length and CPT criteria or not, which occurred in 80% of EUS-LB, 100% of PC-LB, and 98% of TJ-LB cases.

While recognizing the heterogeneity of most comparative studies, Chandan et al.^[44]^ conducted a meta-analysis including five studies and found that EUS-LB was associated with less pain, shorter hospital stays, and a diagnostic yield comparable to that of PC-LB (88.3% *vs*. 98.6%), despite the greater specimen adequacy obtained with the PC method.

These results are comparable to those obtained in our comparative analysis of EUS-LB *versus* PC-LB *versus* TY-LB in terms of diagnostic yield (85% *vs*. 92% *vs*. 76%), mean recovery time (1 hour *vs*. 2–4 hours *vs*. 24 hours), and AE (4% *vs*. 10% *vs*. 15%). Although LSL was higher when using PC-LB, the number of CPT and specimen adequacy was lower than in other studies using any of the three methods.

EUS-LB and PC-LB were compared in four prospective randomized controlled studies, which were analyzed in a recent meta-analysis (total 258 patients) confirming the similarity of the two methods in terms of adequacy diagnostic and AE.^[45]^ None of them include the TJ-LB route in the comparison.

There are only two American publications comparing the costs of EUS-LB and PC-LB^[22,30]^; however, none of them consider indirect costs (such as recovery time) or analyze the costs of TJ-LB. Bang et al. compare the total mean cost of EUS-LB with PC-LB, concluding that PC-LB costs are significantly lower.^[22]^ A review by Mony et al. attempted to compare between the EUS-LB and PC-LB in terms cost-effectiveness in patients with MASLD.^[30]^ Considering that EUS-LB strategy allows initial endoscopic screening for esophageal varices in patients with cirrhosis, EUS-LB proved to be more cost-effective. In our cost-effectiveness analysis, direct and indirect costs were calculated, the main indications of LB were included, and EUS-LB was compared with both traditional LB methods. According to our results, EUS-LB should be selected when the patient has an indication for EUS for any reason, in addition to LB and when PC-LB is contraindicated. In all other situations, PC-LB is less costly and more effective, including in cases of cholestasis, in which MRCP and PC-LB should be considered before EUS-LB in terms of cost utility, unless the patient is unable to undergo MRCP. Our study also probes EUS-LB to be more cost-effective than traditional TJ-LB. The sensitivity analysis confirms higher cost-utility of EUS-LB in those clinical scenarios, despite possible changes in the geographical environment and/or time moment.

According to our findings, EUS-LB should be the preferred strategy in the guideline recommendations for the management of patients with liver disease in two clinical scenarios: when there is concomitant indication for both EUS and LB and when PC-LB is contraindicated (due to ascites or coagulopathy) or cannot be performed (due to anxiety, lack of cooperation or overweight). Furthermore, the EUS-LB method shall be chosen in cases of cholestasis, in order to exclude biliary obstruction, especially when an MRCP cannot be performed (due to pacemaker or claustrophobia). Therefore, due to its effectiveness, good safety profile, and lower associated cost, EUS-LB can substitute TJ-LB in the algorithms and consensus documents, except in cases where EUS-LB cannot be performed (due to lack of availability, certain gastrointestinal surgeries, or contraindication for deep sedation).

Although our study was prospective and multicenter, a limitation to consider is the retrospective selection of the two LB cohorts using traditional methods. However, the randomized inclusion in the case of PC-LB and systematic inclusion of all available TJ-LB in the study time period may have minimized this intervention bias.^[46]^ We chose the use of a cytologic, rather than histologic, puncture needle in the case of EUS-LB because of its greater availability at any center, flexibility to puncture the LHL from the duodenum, lower cost, and related complications, and especially because of its proven efficacy in multiple studies of EUS-LB.^[15,24,40]^ Nevertheless, the use of new histological needles has the potential to improve our results in terms of sample fragmentation and adequacy, reinforcing the role of EUS-LB in the diagnosis of liver diseases.^[16,25,35]^ Our cost-effectiveness study was performed with a Spanish public health system perspective. Therefore, the replicability to private health systems such as the American is debatable due to the cost variability between public and private health systems. However, the sensitivity analysis confirms that our methodology is replicable and scalable to other public health centers in Spain and Europe.

In conclusion, our results are consistent with those of other studies in that EUS-LB is a good alternative to traditional LB methods as it provides similar efficacy, a shorter recovery time, and a better safety profile. Although the collected specimen is usually fragmented, its diagnostic yield is satisfactory when examined by expert pathologists. This is particularly relevant for MASLD, one of the main indications for LB. EUS-LB is the most cost-effective option when both LB and EUS are indicated, in cases of cholestasis as an alternative to MRCP, aiming to rule out biliary obstruction preceding the LB, and when the PC route is not recommended. In fact, EUS should be considered in the guidelines and consensus documents as the best LB method for specific indications in patients with liver disease.

## Source of Funding

This work was supported, in part, by the Foundation of the Spanish Society of Digestive Endoscopy (FSEED).

## Conflicts of Interest

None of the authors have a conflict of interest.

## Ethical Approval

This study was reviewed and approved by the Ethics Committee from Hospital Universitario 12 de Octubre de Madrid (reference number 17/092) and the local ethics committees of each participating center.

## Informed Consent

All patients provided written informed consent to participate in this study.

## Author Contributions

T. Álvarez-Nava and M. Pérez-Carreras conceived and coordinated the study, performed EUS–guided liver biopsy, and wrote the original draft. C. Ibarrola and Y. Rodríguez-Gil contributed to the intellectual work and performed the histopathological analysis. F. de la Morena, J. Díaz-Tasende, and C. de la Serna contributed to patient enrollment and performance of EUS–guided liver biopsy. C. Martin-Arriscado contributed to the statistical analysis. A. Martín and I. Fernández participated in the inclusion of patients and performance of percutaneous liver biopsy. A. Sánchez performed transjugular liver biopsy.

## Data Availability Statement

All data relevant to the study are included in the article.
